# General Rules for Optimal Codon Choice

**DOI:** 10.1371/journal.pgen.1000556

**Published:** 2009-07-10

**Authors:** Ruth Hershberg, Dmitri A. Petrov

**Affiliations:** Department of Biology, Stanford University, Stanford, California, United States of America; University of Arizona, United States of America

## Abstract

Different synonymous codons are favored by natural selection for translation efficiency and accuracy in different organisms. The rules governing the identities of favored codons in different organisms remain obscure. In fact, it is not known whether such rules exist or whether favored codons are chosen randomly in evolution in a process akin to a series of frozen accidents. Here, we study this question by identifying for the first time the favored codons in 675 bacteria, 52 archea, and 10 fungi. We use a number of tests to show that the identified codons are indeed likely to be favored and find that across all studied organisms the identity of favored codons tracks the GC content of the genomes. Once the effect of the genomic GC content on selectively favored codon choice is taken into account, additional universal amino acid specific rules governing the identity of favored codons become apparent. Our results provide for the first time a clear set of rules governing the evolution of selectively favored codon usage. Based on these results, we describe a putative scenario for how evolutionary shifts in the identity of selectively favored codons can occur without even temporary weakening of natural selection for codon bias.

## Author Summary

Codon bias is a long recognized and long studied biological phenomenon. Yet several basic questions regarding codon usage remain unresolved. Here, we address one such basic open question: the identity of the codons that are favoured by selection for translation accuracy and efficiency varies greatly and, at first glance, idiosyncratically among genomes. What are the rules governing the identity of favoured codons in the different genomes? We systematically identified the optimal codons of 675 bacteria, 52 archea, and 10 fungi. Using these data, we show that universally across all bacteria, archea, and fungi the identity of the favoured codons tracks the nucleotide content of the genome as a whole. Once the effect of nucleotide content on selectively favored codon choice is taken into account, additional, until now unknown, universal amino acid specific rules governing the identity of selectively favored codons become apparent. Finally, we use our findings to offer a plausible scenario as to how the identity of optimal codons can shift between genomes by tracking the nucleotide patterns of the genome and without necessitating a reduction in selection.

## Introduction

The genetic code is redundant with most amino acids encoded by several synonymous codons. In many genomes, some codons are favored over others by selection likely because they are translated more efficiently and accurately [1]–[5]. The selectively favored codons tend to correspond to the most highly expressed tRNAs [6]–[9]. Selection for the use of favored codons should be stronger for genes that are more highly expressed. For this reason, highly expressed genes such as ribosomal genes or translation elongation factors use favored codons almost exclusively and exhibit very high levels of codon bias [6], [10]–[13]. In contrast, the identity of the codons used by many genes that are not highly expressed may be determined to a large extent by the nucleotide substitution patterns of the genome that are unrelated to natural selection at the level of translation. Previous studies have demonstrated that the overall codon usage patterns of genomes can be predicted based solely on the nucleotide composition of their intergenic regions [14],[15]. Such studies were interpreted as showing that for most genes selection at the level of translation is only secondary in determining codon usage, as it is too weak to counteract the effects of biases in the patterns of nucleotide substitution that are experienced by the genome in general [14],[15].

The identity of selectively favored codons varies among organisms [16]–[18]. For example, the favored codon for leucine in *Escherichia coli* and *Drosophila melanogaster* is CTG, in *Bacillus subtilis* TTA, in *Saccharomyces cerevisiae* TTG, and in *Saccharomyces pombe* CTT [18]. The rules governing the identities of favored codons in different organisms remain entirely obscure.

One possibility is that the optimal codons are chosen randomly in evolution in a process akin to the frozen accident hypothesized to have occurred in the evolution of the genetic code [19]. However, there are some serious difficulties with this possibility. First, some optimal codon choices appear highly structured and counterintuitive. For instance, in Drosophila all optimal codons are G or C ending (majority are C ending) while the genome is ∼65% AT rich on average [17]. Even more problematic is the observation that the identity of optimal codons shifts in evolution quite readily. This implies that the frozen accidents of optimal codon choice can become “unfrozen” at times and then after a period of time become frozen again but in a new state. Such shifts would seem to require long periods of weak selection given that they would require a large number of genes to change at a large number of sites seemingly against the pressure of natural selection [1].

One difficulty in gaining insight into this problem is that only few metazoans have clear selection-driven codon bias and the identity of favored codons in other organisms such as bacteria, archea and fungi have not yet been determined. Here we identify the favored codons in 675 fully sequenced bacterial genomes, 52 archeal genomes and 10 fungal genomes (Text S1, S2, S3). We demonstrate that, unlike in Drosophila, the identities of favored codons in bacteria, archea, and fungi correspond to the nucleotide content of the intergenic regions of each genome. Thus, GC rich organisms tend to have GC rich favored codons while AT rich organisms tend to have AT rich favored codons. This indicates that, unlike previously suggested, selection is not secondary in determining the codon usage patterns of genomes. Rather, selection consistently acts in the same direction as the nucleotide substitution biases that determine the nucleotide content of genomes in general. We further use the data in bacteria to demonstrate that once nucleotide substitution patterns are taken into account additional amino-acid specific rules determining the identity of favored codons become apparent. Finally, our findings allow us to suggest a possible mechanism by which the identity of favored codons can change between genomes without necessitating prolonged periods of weak selection on the efficiency and accuracy of translation.

## Results/Discussion

### Identification of selectively favored codons

We begin by considering bacterial genomes. A straightforward and widely used way to identify the favored codons is to ask which of the codons encoding a particular amino acid increase in frequency as genes become more biased in the choice of codons overall [13],[17],[20],[21]. Following this reasoning, for each of the 675 bacteria, we calculated the overall degree of codon bias for each gene using the effective number of codons (Nc) ([22], Materials and Methods). Nc measures codon bias of a gene across all codon families without making any assumptions regarding the identity of optimal codons. Values of Nc range between 20, for extremely biased genes that use only one codon per amino acid, to 61, for genes that use all synonymous codons equally. A version of Nc, Nc' was suggested by Novembre [23]. Nc' takes into account and adjusts for background nucleotide composition. The intent of Nc' is to define codons that are used unusually frequently given the background GC content of the considered protein coding sequence [23]. For each of the 18 amino acids that are encoded by more than a single codon, we examined the correlation between the frequency of each of its synonymous codons in a gene and the Nc' of the gene. For each amino acid we identified the most favored (optimal) codon defined as the codon that showed both the strongest and statistically significant positive Spearman correlation with the overall level of codon bias (*P*≤*0.05/n*, where n is the number of codons encoding the amino acid in question, Materials and Methods). For some amino acids in some organisms we could find no favored codons. The identities of the identified optimal codons, for each of the 18 amino acids, in each of the 675 bacteria are summarized in Table S1.

Codon bias can be the result not only of selection but also of variation in the patterns of nucleotide substitution. Thus, in order to demonstrate that the codons identified by our procedure are in fact selectively favored, it is necessary to show that variation in codon bias among genes within most genomes cannot be explained without the involvement of selection. To do so, we conducted two tests. First, we examined whether the most codon biased (MCB) genes are the most highly expressed genes. Specifically, we asked whether ribosomal genes and translation elongation factors, which are often among the highest expressed genes [24],[25], are statistically significantly (*P*<0.05) over-represented among the 100 MCB genes in each genome (Materials and Methods). We found that for 658 of the 675 bacterial genomes studied here this is indeed the case (Table S2). For most of the bacteria in the study the P-value was much lower than 0.05 (Table S2). This test might be weakened by imperfect annotations in some genomes. Nevertheless, it does show that for the vast majority of bacteria the MCB genes are likely under the strongest selection for optimal codon usage.

In order to further demonstrate that codon bias in these genomes is not entirely due to variability in patterns of nucleotide substitutions unrelated to translational selection, we extracted in each genome the first 100 fourfold and twofold degenerate codons of each coding sequence. We then replaced the third codon positions of these coding segments (CS) with 100 randomly selected nucleotides from the intergenic sequences adjacent to them, while maintaining to identity of the encoded amino acids. This resulted in a set of intergenic control coding segment (ICCS) that maintain the protein sequences and nucleotide content patterns of the genome but remove the effects of selection on synonymous sites that we expect to see in the CS. We calculated the level of codon bias of each of the ICCS and each of the CS and examined for each genome whether the 100 most codon biased CS are significantly more biased than the 100 most codon biased ICCS (*P*≤0.05, using a one-tailed Wilcoxon test). We found that this is indeed the case for all but one of the 675 bacteria examined. As in the previous test *P*-values were always much smaller than 0.05 (Table S2). This further suggests that for the vast majority of organisms the optimal codons we identified are indeed likely to be selectively favored.

### Identity of optimal codons tracks genome nucleotide content in all three kingdoms of life

**Figure 1:**
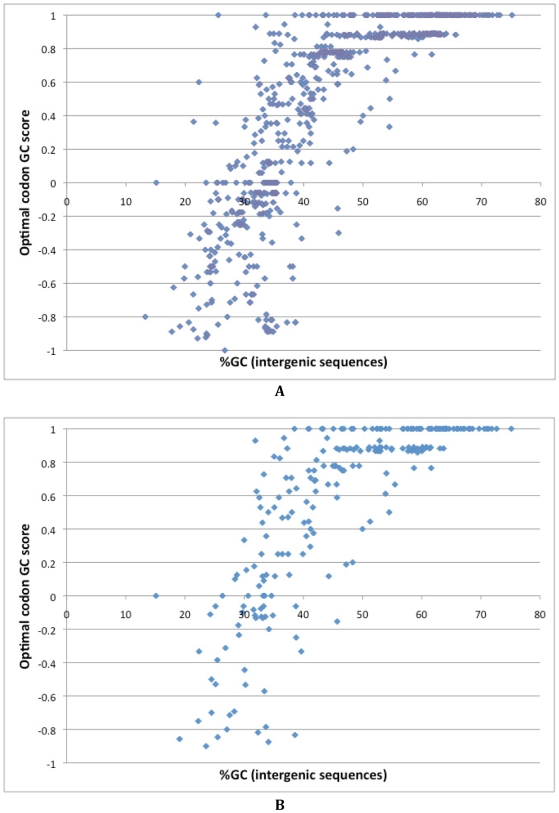
GC richness of optimal codons correlates with intergenic GC content in bacteria. The most GC-rich codons in each codon family received a score of 1, the most AT rich codons in each codon family received a score of −1. For Arginine and Leucine codons of intermediate GC content received a score of 0. For each genome the GC scores of the optimal codons were summed and divided by the number of codon-families for which an optimal codon was identified. Thus an organism that has only GC-rich optimal codons received a score of 1, while an organism that uses only AT-rich optimal codons received a −1. These scores are plotted against the intergenic GC content. (A) All bacteria are included, and (B) one bacteria selected at random from each genus.

An examination of the identified optimal codons (Table S1) led us to realize that there appears to be a relationship between the identity of optimal codons and intergenic GC content. To examine this relationship systematically we classified the codons in each codon family into the most GC rich, the most AT rich, and those with intermediate GC content (such codons exist only for Leucine and Arginine). We gave a score of 1 to each GC rich codon, a score of −1 to each AT rich codon and a score of 0 to the intermediate codons (Table S3). For each genome we summed the scores of its optimal codons and divided the sum by the number of codon-families for which we could identify the optimal codon. Thus an organism that has only GC-rich optimal codons will receive a score of 1 while an organism that uses only AT-rich optimal codons will receive a −1. We plotted these scores against the intergenic GC contents of the genomes (Figure 1A) and found a clear correlation between the optimal codon GC score and intergenic GC content (*r_spearman_* = 0.88, n = 675, *P*≪0.00001). In order to eliminate the possible effects of close taxonomic relationships between some of the analyzed bacteria, we repeated this analysis after randomly selecting a single representative from each bacterial genus. The correlation between the optimal codon GC score and intergenic GC content (Figure 1B) remains highly significant (*r_spearman_* = 0.84, n = 263, *P*≪0.00001).

**Figure 2:**
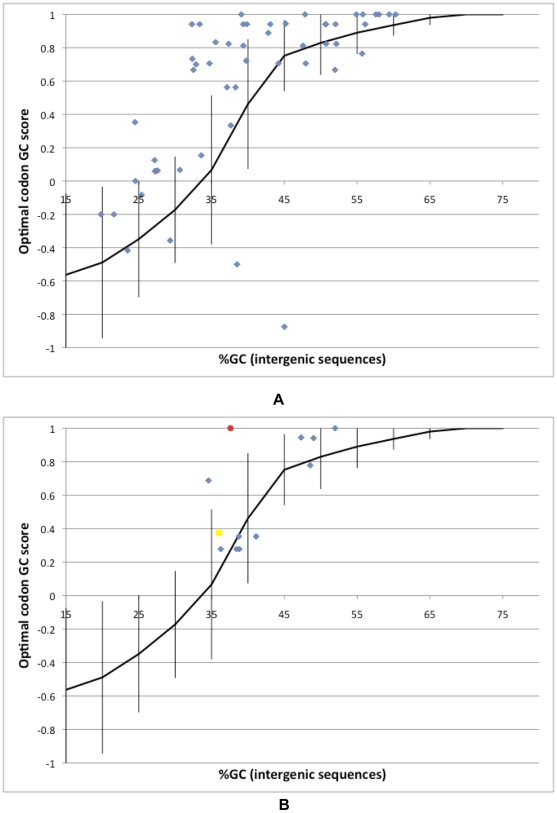
GC richness of optimal codons correlates with intergenic GC content in archea and fungi. The optimal codon GC score is plotted against intergenic GC content. (A) archea. (B) fungi (blue diamonds), *D. melanogaster* (red circle), and *C. elegans* (yellow square). In both (A) and (B) we include for comparison a trend line reflecting the relationship between the optimal codon GC score and intergenic GC in bacteria. To create this trend line bacterial genomes were binned in increments of 5% by their intergenic GC contents (±2.5% centered around the point indicated on the x-axis). The y-value of each point represents the average of the optimal codon GC scores of the corresponding bacteria, while the error bars represent the standard deviations of these values.

We repeated this analysis for the 52 archea (Figure 2A and Table S4) and the 10 fungi (Figure 2B and Table S5). We found that for both of these groups there are similar correlations between the intergenic GC content and the optimal codon GC score (*r_spearman_* = 0.73, n = 52, *P*<0.00001 for archea, *r_spearman_* = 0.74, n = 10, *P*≤0.02023 for fungi). Vicario *et al.* [17] found that *D. melanogaster* has only GC-rich optimal codons even though the nucleotide substitution patterns of its genome tend towards AT. When we plot the optimal codon score for *D. melanogaster* (calculated based on the optimal codons identified in Vicaro et al. [17]) against the background GC content of *D. melanogaster* (estimated in the same paper, based on the sequences of short introns [17], Figure 2B), we find that for its low GC content *Drosophila* appears to be using a higher proportion of GC rich codons than any of the other three groups of organisms. We also analyzed an additional metazoan, *Caenorhabditis elegans*, that has a lower optimal codon GC score and a lower GC content [26] than *D. melanogaster* (Figure 2B). However, there are not enough fully sequenced metazoan genomes with documented selection-driven codon bias to examine the relationship between optimal codon identity and nucleotide content in Metazoa.

It is important to note that there is no *a priori* reason why translationally favored codons should match the nucleotide content of intergenic DNA. Previous studies have demonstrated a relationship between overall codon usage of genomes and their intergenic GC content [14],[15]. Because in these studies little attention was given to the inner-genome variation in the patterns of codon usage, these results were thought to indicate that selection makes only a weak contribution to creating codon biases, and that the major contributor to the codon bias phenomenon are genome-wide nucleotide substitution biases. By identifying optimal codons and showing that their identity also tracks nucleotide content of intergenic regions we demonstrate that it is not that selection weakly affects codon bias, but rather that it appears to be consistently acting in the same direction as the nucleotide substitution biases of genomes.

**Figure 3:**
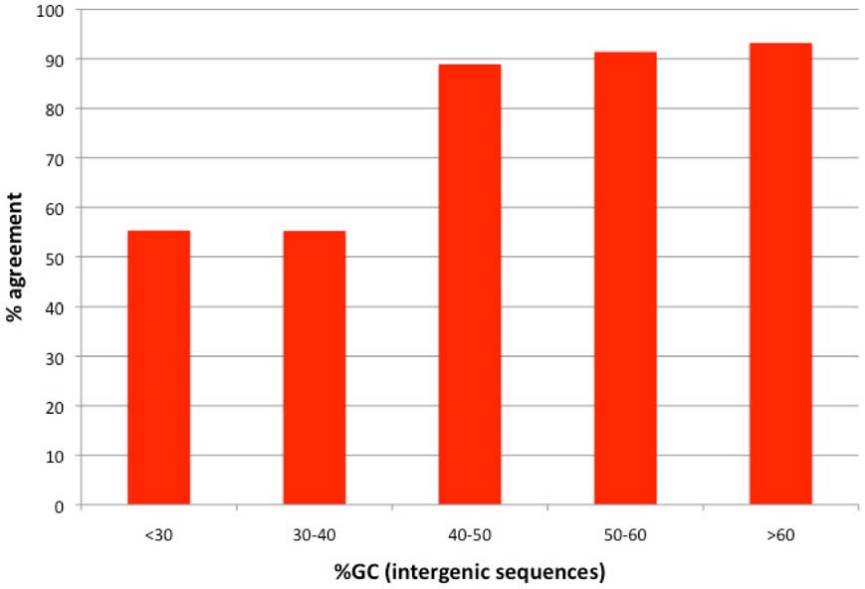
Percentage of agreement in optimal codon identification using Nc' and Nc. Bacteria were divided into 5 groups based on their intergenic GC contents. Cases in which one or both of the methods did not identify any codon as optimal were ignored.

In order to identify optimal codons, we used Nc', a measure of codon bias that corrects for variation in genomic GC content [23]. Given our findings it is possible that by using this method we eliminated some of the signal we'd expect to find. For example, based on our findings we expect that the optimal codons in a GC rich genome should be GC rich. Highly expressed genes will use optimal codons more and will be more codon biased and more GC rich. Nc' is expected to correct some of this effect out even though it is in fact true signal rather than noise. Indeed when we identify optimal codons in bacteria using Nc, rather than Nc' we find an even stronger correlation between the GC richness of optimal codons and the GC richness of intergenic sequences (Figure S1, r_spearman_ = 0.91, n = 675, *P*≪0.00001). Interestingly we find that the same optimal codons are almost always identified using both Nc and Nc' for genomes with intergenic GC contents higher than 40% (Figure 3). However, for genomes with intergenic GC contents lower than 40% the same optimal codon is identified in only ∼50% of cases. In addition we found that our ability to identify optimal codons is much reduced in AT rich genomes. These two findings make sense if selection to use optimal codons is generally weaker for AT rich genomes than for GC rich genomes. Indeed, many of the AT rich bacteria are endosymbionts that are known to be slow growing and in which selection for translation accuracy and efficiency is thought to be weaker [27],[28]. Even if genomes with GC contents below 40%, for which our ability to clearly identify optimal codons appears to be somewhat reduced are removed from consideration, the correlation between intergenic GC content and the optimal codon GC score remains highly significant (r_spearman_ = 0.73, n = 366, *P*≪0.00001). It thus appears that our finding of a relationship between intergenic GC content and the identity of optimal codons is robust to the possible misidentification of optimal codons in the AT rich genomes.

### Additional rules governing the identity of optimal codons

**Figure 4:**
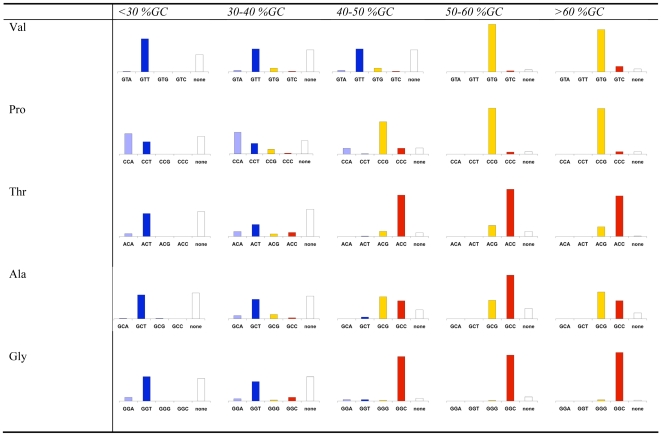
Optimal codon identities in fourfold degenerate codon families. Bacteria were divided based on their intergenic GC contents. For each codon family in each intergenic GC content grouping the small bar graph depicts the percentage of the bacterial groups for which each of the four possible codons is optimal (has the most significant (p≤0.0125) correlation with levels of codon bias, as measured using both Nc and Nc'). Cases in which no optimal codon was found using either Nc or Nc', or in which different optimal codons were identified using the two measures are counted as “none.” The graphs are all scaled to size.

**Figure 5:**
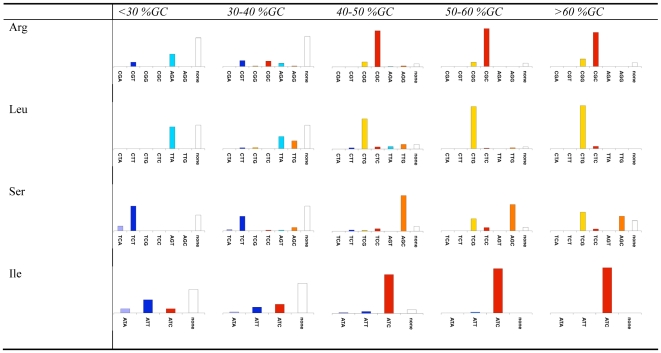
Optimal codon identities in codon families with six or three members. Bacteria were divided based on their intergenic GC contents. For each codon family in each intergenic GC content grouping, the small bar graph depicts the percentage of the bacterial groups for which each of the possible codons is optimal (has the most significant (p≤0.05/n, where n is the number of codons in the codon family) correlation with levels of codon bias, as measured using both Nc and Nc'). Cases in which no optimal codon was found using either Nc or Nc', or in which different optimal codons were identified using the two measures are counted as “none.” The graphs are all scaled to size.

**Figure 6:**
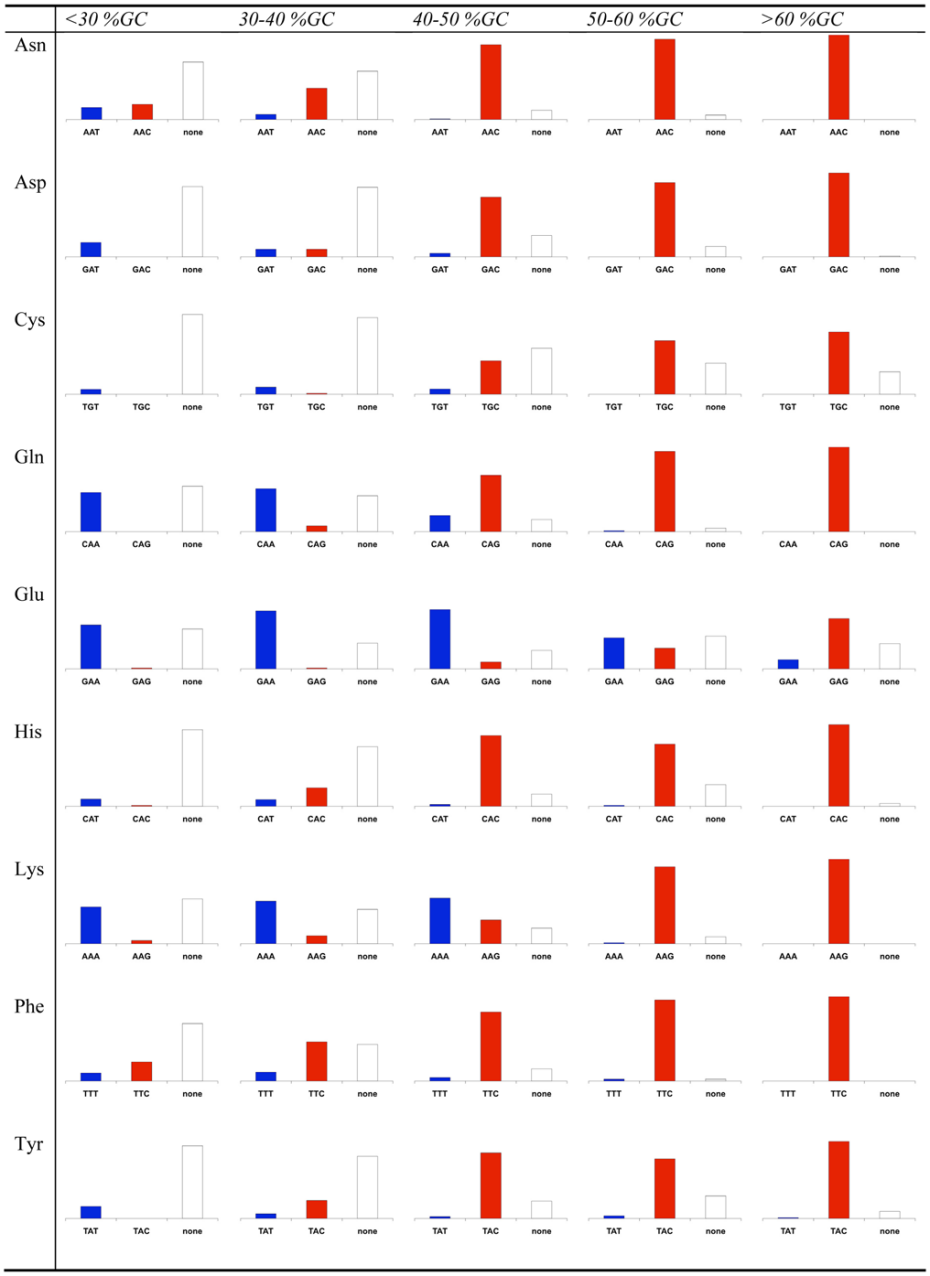
Optimal codon identities in two-fold degenerate codon families. Bacteria were divided based on their intergenic GC contents. For each codon family in each intergenic GC content grouping, the small bar graph depicts the percentage of the bacterial groups for which each of the two possible codons is optimal (has the most significant (p≤0.025) correlation with levels of codon bias, as measured using both Nc and Nc'). Cases in which no optimal codon was found using either Nc or Nc', or in which different optimal codons were identified using the two measures are counted as “none.” The graphs are all scaled to size.

To learn more about the rules governing the identity of optimal codons we split all genomes into five groups based on their intergenic GC content. We summarized the identities of the optimal codons in each group for the fourfold degenerate codon families, the codon families with three or six codons, and the twofold degenerate codon families in Figure 4, Figure 5, and Figure 6 respectively. To be more certain of our assignment of optimal codons, we demanded that the same optimal codon be identified using both correlations with Nc' and correlations with Nc. If for a certain codon family in a certain genome one or both of these correlations resulted in the identification of no optimal codon, or if they both identified different optimal codons we classify the optimal codon as “none”.

Examining these figures allowed us to observe again that GC rich bacteria tend to use GC rich optimal codons while AT rich bacteria tend to use AT rich optimal codons. However, these figures also demonstrate additional rules governing the identity of optimal codons in bacteria. For example, among the fourfold degenerate codons (Figure 4), for high GC organisms, C is strongly preferred over G in the optimal codons of Threonine, and Glycine. At the same time G appears to be preferred over C in the optimal codons of Proline, and Valine. Our results are less clear for AT rich genomes, as in such genomes for more codon families in more organisms we could identify no clear optimal codon. However, in such genomes, T appears to be preferred over A in the optimal codons of all fourfold degenerate codon families other than Proline. Similarly interesting patterns can be seen for codon families with six members (Figure 5). For Leucine, for example, in AT rich genomes the TTA codon is preferred among optimal codons. This makes sense as this is the most AT rich codon encoding Leucine. At the same time, for the optimal codons of GC rich bacteria the CTG codon is strongly preferred over the equally GC rich CTC codon. A similar pattern appears for Arginine. For AT rich genomes the optimal codon is most frequently the most AT rich codon (AGA). However, for GC rich genomes CGC is almost always selected over CGG. Such family specific patterns are intriguing and require further study.

In a previous study [28] Rocha investigated codon bias from the tRNA perspective by analyzing the copy numbers of the tRNAs with different anticodons in different genomes. Surprisingly, he found that the most frequent anticodons remain constant across different genomes and do not change with GC content. Rocha observed that generally in the first anticodon position (which will bind to the third codon position) of twofold-degenerate amino acids, G is always more frequent than A while T is more frequent than C. He therefore expected to observe a preference for C or A in third codon positions of these codon families over G and T [28]. We observe that similarly to other codon families the tendency of organisms to use the more AT rich or GC rich optimal codon out of the two possible twofold degenerate codons depends on intergenic GC content (Figure 6). However, for codon families that can end in either G or A (Gln, Glu and Lys) the shift from using the more AT rich optimal codons to using the more GC rich optimal codons tends to occur at higher GC contents, compared to the codon families that end in either C or T (Asn, Asp, Cys, His, Phe and Tyr). This means that more organisms use the C or A ending codons as expected from Rocha's results.

For many organisms only a single tRNA exists for a certain codon family. It is therefore clear that tRNA modifications and wobble rules are involved in allowing a single tRNA to bind different codons. These wobble rules and modifications may be different in different organisms. Such differences made it difficult for Rocha to define expectations as to which codons would be best recognized by the most frequent anticodons in each organism for codon families with more than two members [28]. We could therefore not compare the results of Rocha to our results for such codon families.

### Shifts in the identity of optimal codons may not require prolonged periods of weakened selection

Our results not only provide a clear set of rules governing the identity of the favored codons, they also provide a possible mechanism by which this identity can shift between organisms. Variation in GC content across genomes implies shifts in nucleotide content. The pattern we found implies that such shifts in nucleotide content are accompanied by shifts in the identity of favored codons. Let us consider a scenario in which a genome begins shifting towards a different global GC content that does not match the GC content of its favored codons. After a while, genes that are not under strong selection at the level of translation will start using codons that correspond to the new GC content of the genome. While, individually these genes may not be expressed highly enough to be under strong selection for the use of favored codons, together they may affect the efficiency of translation substantially. For this reason it may become advantageous for the tRNAs that correspond to these newly frequent codons to increase their expression. While Rocha has shown that the identity of the tRNA with the highest copy number does not tend to change much between bacteria [28], this can be achieved by increasing the transcription of a certain anticodon tRNA, or through regulation of tRNA modifications. Following this increase, the highly expressed genes will be free to start using the codons that correspond more to the GC content of the genome. This will be encouraged by the new pattern of nucleotide substitutions of the genome and should eventually remove the selection for the high expression of the tRNAs that recognize the old favored codons. As a result after a time new favored codons may emerge that correspond to the nucleotide content of the genome. In order to prove such a scenario it will be necessary to carefully examine shifts in nucleotide content and in the identity of optimal codons across a bacterial phylogenetic tree. In such a way it may be possible to ask whether changes in the identity of optimal codons indeed follow changes in nucleotide content. This analysis is beyond the scope of this paper and so it is important to note that the scenario we suggest here for shifts in optimal codon usage is hypothetical. This scenario is intriguing however as, if true, it explains how the identity of favored codons can shift without requiring a prolonged period of weakened selection. Furthermore, this scenario suggests that while selection for the use optimal codons is strongest for a specific set of highly expressed genes, the identity of the optimal codons is in fact determined largely by the majority of genes, on which selection is much weaker.

### Concluding remarks

The codon bias phenomenon has been studied for decades. Yet, basic questions regarding this phenomenon remain unanswered. Here, we provide an insight into one such basic open question: What determines the identity of the codons favored by selection for translation accuracy and efficiency in different genomes. We show that in all three kingdoms of life the identity of the favored codons matches the nucleotide content of the intergenic regions of each genome. Furthermore, once the relationship between the identity of favored codons and nucleotide content is taken into account additional amino-acid specific rules determining the identity of favored codons come to light. We then use our findings to provide a possible answer to a second open question: how can the identity of favored codons shift in evolution and do such shifts require prolonged periods of weakened selection? Our findings allow us to suggest a scenario for shifts in the identity of favored codons that does not require a weakening of selection.

## Materials and Methods

### Data used

The completed genomic sequences and coding sequence annotaions of the 675 bacteria, 52 archea, and 10 fungi were downloaded from the NCBI FTP server. (ftp://ncbi.nlm.nih.gov).

### Calculating the overall codon bias of genes

For each of the fully sequenced bacteria, archea and fungi used in the study (Text S1, S2, S3) we extracted the DNA coding sequences of all the annotated proteins. For each protein in each genome we calculated the effective number of codons (Nc [22]). Nc, measures the overall codon bias of a gene across all codon families [22]. The measure does not make any assumptions regarding the identity of the optimal codons. Values of Nc range between 20, for extremely biased genes that use only one codon per amino acid, to 61, for genes that use all synonymous codons equally [22]. Since the estimation of Nc is problematic for short sequences, we removed from consideration coding sequences shorter than 50 codons. In order to further account for sensitivity to sequence length, we used the version of Nc supplied by Novembre as part of his ENCprime package that corrects for sequence length [23]. Nucleotide content is also expected to affect Nc. We therefore also used a version of Nc, Nc' which was developed by Novembre and which corrects for nucleotide content [23].

### Determining the identity of optimal codons

In order to identify optimal codons for a specific genome we calculated for each codon its frequency within its codon family in all of the annotated coding sequences in each genome. We then calculated the correlation between the frequency of each codon within each gene and the overall codon bias (once using Nc' and once using Nc [23]) of that gene. We removed from consideration genes in which the codon family appeared less than 10 times. The optimal codon for each codon family was defined as the codon that showed the strongest and significant negative correlation with the Nc or Nc' of the gene. To be considered significant a correlation had to have a *P*-value smaller or equal to 0.05/n, where n is the number of codons in the codon family. In such a way we correct for the fact that we performed more comparisons for more degenerate codon families. Spearman correlations were performed using the R statistical package.

### Selecting a single representative from each bacterial genus

In order to randomly select a single member of each bacterial genus, bacteria sharing a genus name (i.e. *Escherichia*, or *Mycobacterium*) were grouped and a single member of each group was randomly selected.

### Testing whether ribosomal genes and translation elongation factors are overrepresented among the 100 most biased genes

For each genome, we counted how many of the 100 most biased (lowest Nc) genes are annotated as “ribosomal” or “elongation factor”. We then randomly selected 100 of the remaining genes in the genome and counted how many of these random genes are annotated as ribosomal genes or elongation factors. We repeated this randomization 1000 times and calculated the *P*-value that tells us in how many of these random samples does an annotation of “ribosomal” or “elongation factor” appear as often or more often than for the most biased genes. We say that ribosomal genes and elongation factors are significantly over represented among the 100 most biased genes if this *P*-value is lower or equal to 0.05.

### Creating sets of intergenic control coding sequences

To create the intergenic control coding sequences (ICCS) we used the following strategy for each of the 675 genomes. I) We extracted the first 100 four-fold degenerate and two-fold degenerate codons of each protein coding gene. We removed from consideration genes that had less than 100 two-fold and four-fold degenerate codons. II) For each protein coding gene we extracted its two adjacent intergenic sequences. We concatenated both adjacent intergenic sequences (the 5′ and the 3′ intergenic sequences) and selected a 100 base pair segment of this sequence at random. We shuffled the order of the nucleotides of these intergenic segments randomly. We removed intergenic regions shorter than 50 bases and if for a gene there was not at least 100 bases of adjacent intergenic region, we removed that gene from consideration. III) We created ICCS using the real coding sequences as a backbone and replacing the third codon positions, based on the shuffled adjacent intergenic sequences, while maintaining the encoded protein sequence. For example if in the real protein at the tenth position we have a Valine encoded by the four-fold degenerate codon GTG and the shuffled segment of the adjacent intergenic sequence has a T in the tenth position, our ICCS will have a GTT in the tenth codon position. In the case of a two-fold degenerate codon such as the Lysine codons AA(A/G), we selected AAG if the corresponding intergenic position contained either a G or a C and AAA if the corresponding intergenic position contains an A or a T.

At the end of this process we obtained for each genome two sets of coding segments of a consistent length; the “real” coding sequences (CS) and the ICCS. Both of these encode exactly the same proteins. The third codon positions of the ICCS reflect the composition of the real gene's adjacent intergenic regions.

## Supporting Information

Figure S1 Stronger correlation between optimal codon GC score and intergenic GC contents when identifying optimal codons based on correlations with Nc rather than Nc'. The most GC-rich codons in each codon family received a score of 1, the most AT rich codons in each codon family received a score of −1. For Arginine and Leucine codons of intermediate GC content received a score of 0. For each genome the GC scores of the optimal codons (identified using Nc) were summed and divided by the number of codon-families for which an optimal codon was identified. Thus an organism that has only GC-rich optimal codons received a score of 1 while an organism that uses only AT rich optimal codons received a −1. These scores are plotted against the intergenic GC content.
(3.00 MB TIF)

Table S1 Optimal codons identified in bacteria.
(0.25 MB XLS)

Table S2 Results of tests for involvement of selection.
(0.15 MB XLS)

Table S3 Codon GC scores.
(0.08 MB DOC)

Table S4 Optimal codons identified in archea.
(0.03 MB XLS)

Table S5 Optimal codons identified in fungi.
(0.02 MB XLS)

Text S1 Bacteria used in this study.
(0.38 MB DOC)

Text S2 Fungi used in this study.
(0.04 MB DOC)

Text S3 Archea used in this study.
(0.08 MB DOC)
