# Supplementary figures and images for: General Rules for Optimal Codon Choice
Source: PLoS Genet. 2009 Jul 10;5(7):e1000556. doi: 10.1371/journal.pgen.1000556 (PMC2700274; doi:10.1371/journal.pgen.1000556)

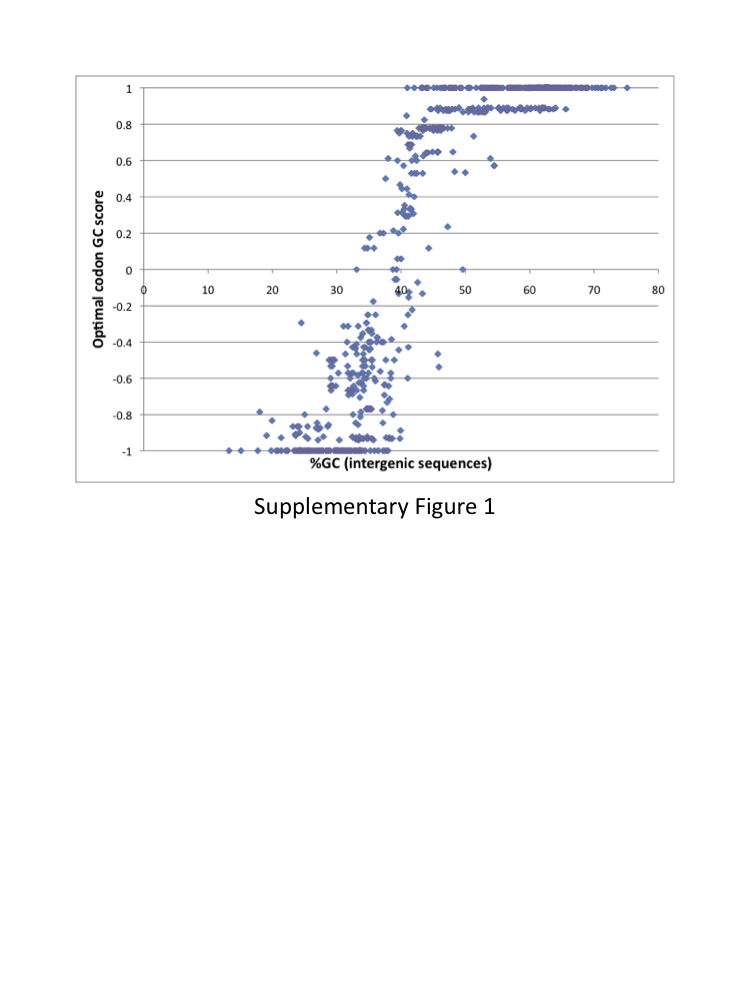

Supplement: Figure S1 — Stronger correlation between optimal codon GC score and intergenic GC contents when identifying optimal codons based on correlations with Nc rather than Nc'. The most GC-rich codons in each codon family received a score of 1, the most AT rich codons in each codon family received a score of −1. For Arginine and Leucine codons of intermediate GC content received a score of 0. For each genome the GC scores of the optimal codons (identified using Nc) were summed and divided by the number of codon-families for which an optimal codon was identified. Thus an organism that has only GC-rich optimal codons received a score of 1 while an organism that uses only AT rich optimal codons received a −1. These scores are plotted against the intergenic GC content. (3.00 MB TIF) [file pgen.1000556.s001.tif]
