# Supplementary material for: General Rules for Optimal Codon Choice
Source: PLoS Genet. 2009 Jul 10;5(7):e1000556. doi: 10.1371/journal.pgen.1000556 (PMC2700274; doi:10.1371/journal.pgen.1000556)
Supplement: Table S3 — Codon GC scores. (0.08 MB DOC) [file pgen.1000556.s004.doc]

**Supplementary Table 3**. Codon GC scores

| *Amino Acid* | *Codon* | *GC score* |
| --- | --- | --- |
| ala | GCA | -1 |
| ala | GCC | 1 |
| ala | GCG | 1 |
| ala | GCT | -1 |
| arg | AGA | -1 |
| arg | AGG | 0 |
| arg | CGA | 0 |
| arg | CGC | 1 |
| arg | CGG | 1 |
| arg | CGT | 0 |
| asn | AAC | 1 |
| asn | AAT | -1 |
| asp | GAC | 1 |
| asp | GAT | -1 |
| cys | TGC | 1 |
| cys | TGT | -1 |
| gln | CAA | -1 |
| gln | CAG | 1 |
| glu | GAA | -1 |
| glu | GAG | 1 |
| gly | GGA | -1 |
| gly | GGC | 1 |
| gly | GGG | 1 |
| gly | GGT | -1 |
| his | CAC | 1 |
| his | CAT | -1 |
| ile | ATA | -1 |
| ile | ATC | 1 |
| ile | ATT | -1 |
| leu | CTA | 0 |
| leu | CTC | 1 |
| leu | CTG | 1 |
| leu | CTT | 0 |
| leu | TTA | -1 |
| leu | TTG | 0 |
| lys | AAA | -1 |
| lys | AAG | 1 |
| phe | TTC | 1 |
| phe | TTT | -1 |
| pro | CCA | -1 |
| pro | CCC | 1 |
| pro | CCG | 1 |
| pro | CCT | -1 |
| ser | AGC | 1 |
| ser | AGT | -1 |
| ser | TCA | -1 |
| ser | TCC | 1 |
| ser | TCG | 1 |
| ser | TCT | -1 |
| thr | ACA | -1 |
| thr | ACC | 1 |
| thr | ACG | 1 |
| thr | ACT | -1 |
| tyr | TAC | 1 |
| tyr | TAT | -1 |
| val | GTA | -1 |
| val | GTC | 1 |
| val | GTG | 1 |
| val | GTT | -1 |
