# Supplementary material for: General Rules for Optimal Codon Choice
Source: PLoS Genet. 2009 Jul 10;5(7):e1000556. doi: 10.1371/journal.pgen.1000556 (PMC2700274; doi:10.1371/journal.pgen.1000556)
Supplement: Text S1 — Bacteria used in this study. (0.38 MB DOC) [file pgen.1000556.s007.doc]

**Text S1.** Bacteria used in this study

| Buchnera_aphidicola_Cc_Cinara_cedri |
| --- |
| Wigglesworthia_brevipalpis |
| Candidatus_Phytoplasma_mali |
| Buchnera_aphidicola |
| Buchnera_sp |
| Mycoplasma_penetrans |
| Candidatus_Blochmannia_floridanus |
| Buchnera_aphidicola_Sg |
| Mycoplasma_pulmonis |
| Candidatus_Blochmannia_pennsylvanicus_BPEN |
| Mycoplasma_mobile_163K |
| Mycoplasma_hyopneumoniae_232 |
| Mycoplasma_capricolum_ATCC_27343 |
| Ehrlichia_ruminantium_Gardel |
| Ehrlichia_ruminantium_Welgevonden_UPSA |
| Mycoplasma_hyopneumoniae_J |
| Aster_yellows_witches-broom_phytoplasma_AYWB |
| Clostridium_perfringens_ATCC_13124 |
| Borrelia_afzelii_PKo |
| Mycoplasma_mycoides |
| Mycoplasma_hyopneumoniae_7448 |
| Ureaplasma_urealyticum |
| Ureaplasma_parvum_serovar_3_ATCC_27815 |
| Borrelia_burgdorferi |
| Clostridium_botulinum_B_Eklund_17B |
| Ehrlichia_canis_Jake |
| Clostridium_perfringens |
| Clostridium_botulinum_A_ATCC_19397 |
| Clostridium_perfringens_SM101 |
| Clostridium_difficile_630 |
| Clostridium_botulinum_A_Hall |
| Clostridium_botulinum_A |
| Clostridium_botulinum_A3_Loch_Maree |
| Borrelia_garinii_PBi |
| Clostridium_botulinum_B1_Okra |
| Onion_yellows_phytoplasma |
| Prochlorococcus_marinus_MIT_9515 |
| Clostridium_botulinum_F_Langeland |
| Rickettsia_typhi_wilmington |
| Candidatus_Vesicomyosocius_okutanii_HA |
| Rickettsia_prowazekii |
| Prochlorococcus_marinus_MIT_9301 |
| Prochlorococcus_marinus_MED4 |
| Ehrlichia_chaffeensis_Arkansas |
| Campylobacter_hominis_ATCC_BAA-381 |
| Fusobacterium_nucleatum |
| Borrelia_turicatae_91E135 |
| Prochlorococcus_marinus_MIT_9215 |
| Prochlorococcus_marinus_MIT_9312 |
| Arcobacter_butzleri_RM4018 |
| Campylobacter_jejuni_81-176 |
| Lawsonia_intracellularis_PHE_MN1-00 |
| Candidatus_Carsonella_ruddii_PV |
| Campylobacter_jejuni_81116 |
| Prochlorococcus_marinus_AS9601 |
| Clostridium_beijerinckii_NCIMB_8052 |
| Mycoplasma_arthritidis_158L3_1 |
| Clostridium_tetani_E88 |
| Mycoplasma_synoviae_53 |
| Candidatus_Sulcia_muelleri_GWSS |
| Campylobacter_jejuni |
| Mesoplasma_florum_L1 |
| Mycoplasma_agalactiae_PG2 |
| Clostridium_novyi_NT |
| Baumannia_cicadellinicola_Homalodisca_coagulata |
| Clostridium_acetobutylicum |
| Francisella_tularensis_novicida_U112 |
| Candidatus_Pelagibacter_ubique_HTCC1062 |
| Campylobacter_jejuni_RM1221 |
| Orientia_tsutsugamushi_Ikeda |
| Campylobacter_fetus_82-40 |
| Francisella_philomiragia_ATCC_25017 |
| Clostridium_kluyveri_DSM_555 |
| Staphylococcus_epidermidis_ATCC_12228 |
| Parachlamydia_sp_UWE25 |
| Staphylococcus_epidermidis_RP62A |
| Campylobacter_jejuni_doylei_269_97 |
| Flavobacterium_johnsoniae_UW101 |
| Mycoplasma_gallisepticum |
| Rickettsia_canadensis_McKiel |
| Flavobacterium_psychrophilum_JIP02_86 |
| Staphylococcus_haemolyticus |
| Francisella_tularensis_holarctica |
| Acholeplasma_laidlawii_PG_8A |
| Candidatus_Ruthia_magnifica_Cm_Calyptogena_magnifica_ |
| Staphylococcus_aureus_N315 |
| Staphylococcus_aureus_NCTC_8325 |
| Staphylococcus_aureus_USA300 |
| Staphylococcus_saprophyticus |
| Staphylococcus_aureus_Mu50 |
| Staphylococcus_aureus_Mu3 |
| Staphylococcus_aureus_Newman |
| Staphylococcus_aureus_MW2 |
| Staphylococcus_aureus_JH1 |
| Staphylococcus_aureus_JH9 |
| Staphylococcus_aureus_COL |
| Staphylococcus_aureus_aureus_MRSA252 |
| Staphylococcus_aureus_RF122 |
| Francisella_tularensis_WY96-3418 |
| Staphylococcus_aureus_aureus_MSSA476 |
| Francisella_tularensis_FSC_198 |
| Francisella_tularensis_holarctica_FTA |
| Francisella_tularensis_tularensis |
| Francisella_tularensis_holarctica_OSU18 |
| Staphylococcus_aureus_USA300_TCH1516 |
| Finegoldia_magna_ATCC_29328 |
| Leptospira_interrogans_serovar_Lai |
| Prochlorococcus_marinus_NATL1A |
| Prochlorococcus_marinus_NATL2A |
| Francisella_tularensis_mediasiatica_FSC147 |
| Leptospira_interrogans_serovar_Copenhageni |
| Trichodesmium_erythraeum_IMS101 |
| Orientia_tsutsugamushi_Boryong |
| Lactococcus_lactis |
| Thiomicrospira_denitrificans_ATCC_33889 |
| Helicobacter_hepaticus |
| Gramella_forsetii_KT0803 |
| Rickettsia_bellii_RML369-C |
| Rickettsia_conorii |
| Rickettsia_akari_Hartford |
| Clostridium_phytofermentans_ISDg |
| Rickettsia_bellii_OSU_85-389 |
| Lactobacillus_acidophilus_NCFM |
| Rickettsia_rickettsii_Sheila_Smith |
| Rickettsia_rickettsii_Iowa |
| Lactococcus_lactis_cremoris_MG1363 |
| Rickettsia_felis_URRWXCal2 |
| Rickettsia_massiliae_MTU5 |
| Alkaliphilus_oremlandii_OhILAs |
| Acinetobacter_baumannii_ACICU |
| Lactobacillus_salivarius_UCC118 |
| Lactobacillus_johnsonii_NCC_533 |
| Helicobacter_pylori_HPAG1 |
| Candidatus_Amoebophilus_asiaticus_5a2 |
| Bacillus_cereus_ZK |
| Bacillus_anthracis_str_Sterne |
| Lactococcus_lactis_cremoris_SK11 |
| Clostridium_thermocellum_ATCC_27405 |
| Sulfurihydrogenibium_YO3AOP1 |
| Cytophaga_hutchinsonii_ATCC_33406 |
| Wolbachia_endosymbiont_of_Brugia_malayi_TRS |
| Prochlorococcus_marinus_CCMP1375 |
| Bacillus_thuringiensis_konkukian |
| Bacillus_anthracis_Ames_0581 |
| Wolbachia_pipientis |
| Helicobacter_pylori_26695 |
| Bacillus_thuringiensis_Al_Hakam |
| Streptococcus_agalactiae_NEM316 |
| Bacillus_cereus_ATCC_10987 |
| Helicobacter_pylori_J99 |
| Bacillus_anthracis_Ames |
| Bacillus_weihenstephanensis_KBAB4 |
| Streptococcus_agalactiae_A909 |
| Acinetobacter_sp_ADP1 |
| Bacillus_cereus_ATCC14579 |
| Caldicellulosiruptor_saccharolyticus_DSM_8903 |
| Helicobacter_acinonychis_Sheeba |
| Thermosipho_melanesiensis_BI429 |
| Acinetobacter_baumannii_AYE |
| Cyanothece_ATCC_51142 |
| Fervidobacterium_nodosum_Rt17-B1 |
| Streptococcus_mutans |
| Streptococcus_agalactiae_2603 |
| Bartonella_bacilliformis_KC583 |
| Elusimicrobium_minutum_Pei191 |
| Listeria_welshimeri_serovar_6b_SLCC5334 |
| Haemophilus_somnus_129PT |
| Leuconostoc_mesenteroides_ATCC_8293 |
| Hydrogenobaculum_Y04AAS1 |
| Pediococcus_pentosaceus_ATCC_25745 |
| Haemophilus_somnus_2336 |
| Oenococcus_oeni_PSU-1 |
| Lactobacillus_gasseri_ATCC_33323 |
| Thermoanaerobacter_X514 |
| Oceanobacillus_iheyensis |
| Legionella_pneumophila_Philadelphia_1 |
| Petrotoga_mobilis_SJ95 |
| Bartonella_henselae_Houston-1 |
| Chlamydophila_felis_Fe_C-56 |
| Thermoanaerobacter_pseudethanolicus_ATCC_33223 |
| Legionella_pneumophila_Paris |
| Treponema_denticola_ATCC_35405 |
| Colwellia_psychrerythraea_34H |
| Prochlorococcus_marinus_MIT_9211 |
| Bartonella_tribocorum_CIP_105476 |
| Alkaliphilus_metalliredigens_QYMF |
| Psychrobacter_cryohalolentis_K5 |
| Enterococcus_faecalis_V583 |
| Legionella_pneumophila_Lens |
| Bacillus_cereus_cytotoxis_NVH_391-98 |
| Chlamydophila_pneumoniae_J138 |
| Mycoplasma_genitalium |
| Streptococcus_gordonii_Challis_substr_CH1 |
| Listeria_innocua |
| Listeria_monocytogenes |
| Vibrio_fischeri_ES114 |
| Helicobacter_pylori_Shi470 |
| Chlamydophila_pneumoniae_TW_183 |
| Chlamydophila_caviae |
| Streptococcus_pneumoniae_CGSP14 |
| Haemophilus_influenzae_86_028NP |
| Lysinibacillus_sphaericus_C3_41 |
| Streptococcus_pneumoniae_G54 |
| Bacteroides_thetaiotaomicron_VPI-5482 |
| Legionella_pneumophila_Corby |
| Actinobacillus_pleuropneumoniae_serovar_7_AP76 |
| Streptococcus_pneumoniae_R6 |
| Listeria_monocytogenes_4b_F2365 |
| Wolbachia_endosymbiont_of_Drosophila_melanogaster |
| Haemophilus_influenzae |
| Streptococcus_pyogenes_MGAS6180 |
| Pseudoalteromonas_haloplanktis_TAC125 |
| Bartonella_quintana_Toulouse |
| Leuconostoc_citreum_KM20 |
| Streptococcus_pyogenes_MGAS10750 |
| Chlamydophila_pneumoniae_CWL029 |
| Streptococcus_pyogenes_MGAS10394 |
| Streptococcus_pyogenes_MGAS10270 |
| Streptococcus_pyogenes_MGAS5005 |
| Mycoplasma_pneumoniae |
| Actinobacillus_pleuropneumoniae_L20 |
| Chlamydophila_pneumoniae_AR39 |
| Streptococcus_thermophilus_CNRZ1066 |
| Streptococcus_thermophilus_LMG_18311 |
| Streptococcus_pyogenes_MGAS315 |
| Streptococcus_pyogenes_MGAS9429 |
| Streptococcus_pneumoniae_D39 |
| Bacteroides_fragilis_YCH46 |
| Streptococcus_pneumoniae_Hungary19A_6 |
| Streptococcus_sanguinis_SK36 |
| Streptococcus_pyogenes_SSI-1 |
| Streptococcus_pyogenes_MGAS8232 |
| Bacteroides_vulgatus_ATCC_8482 |
| Mannheimia_succiniciproducens_MBEL55E |
| Streptococcus_pyogenes_MGAS2096 |
| Pasteurella_multocida |
| Streptococcus_pyogenes_M1_GAS |
| Campylobacter_concisus_13826 |
| Acinetobacter_baumannii_ATCC_17978 |
| Lactobacillus_helveticus_DPC_4571 |
| Psychrobacter_arcticum_273-4 |
| Streptococcus_pneumoniae_TIGR4 |
| Streptococcus_pyogenes_Manfredo |
| Chlamydophila_abortus_S26_3 |
| Lactobacillus_reuteri_F275_Kitasato |
| Haemophilus_ducreyi_35000HP |
| Thiomicrospira_crunogena_XCL-2 |
| Haemophilus_influenzae_PittEE |
| Acinetobacter_baumannii_SDF |
| Psychromonas_ingrahamii_37 |
| Actinobacillus_pleuropneumoniae_serovar_3_JL03 |
| Bacteroides_fragilis_NCTC_9434 |
| Thermoanaerobacter_tengcongensis |
| Sulfurovum_NBC37-1 |
| Streptococcus_thermophilus_LMD-9 |
| Haemophilus_influenzae_PittGG |
| Streptococcus_suis_05ZYH33 |
| Streptococcus_suis_98HAH33 |
| Nostoc_sp |
| Anabaena_variabilis_ATCC_29413 |
| Parabacteroides_distasonis_ATCC_8503 |
| Shewanella_frigidimarina_NCIMB_400 |
| Lactobacillus_sakei_23K |
| Photorhabdus_luminescens |
| Chlamydia_muridarum |
| Nostoc_punctiforme_PCC_73102 |
| Psychrobacter_PRwf-1 |
| Desulfotomaculum_reducens_MI-1 |
| Leptospira_borgpetersenii_serovar_Hardjo-bovis_L550 |
| Marinomonas_MWYL1 |
| Nitratiruptor_SB155-2 |
| Leptospira_borgpetersenii_serovar_Hardjo-bovis_JB197 |
| Actinobacillus_succinogenes_130Z |
| Photobacterium_profundum_SS9 |
| Thermotoga_lettingae_TMO |
| Leptospira_biflexa_serovar_Patoc__Patoc_1__Ames_ |
| Bacillus_pumilus_SAFR-032 |
| Leptospira_biflexa_serovar_Patoc__Patoc_1__Paris_ |
| Chloroherpeton_thalassium_ATCC_35110 |
| Shewanella_woodyi_ATCC_51908 |
| Chlamydia_trachomatis |
| Chlamydia_trachomatis_A_HAR-13 |
| Streptococcus_equi_zooepidemicus_MGCS10565 |
| Bacillus_subtilis |
| Chlorobium_chlorochromatii_CaD3 |
| Carboxydothermus_hydrogenoformans_Z-2901 |
| Chlamydia_trachomatis_L2b_UCH_1_proctitis |
| Chlamydia_trachomatis_434_Bu |
| Dichelobacter_nodosus_VCS1703A |
| Neorickettsia_sennetsu_Miyayama |
| Zymomonas_mobilis_ZM4 |
| Lactobacillus_plantarum |
| Shewanella_halifaxensis_HAW_EB4 |
| Vibrio_parahaemolyticus |
| Shewanella_pealeana_ATCC_700345 |
| Bacillus_licheniformis_DSM_13 |
| Shewanella_putrefaciens_CN-32 |
| Dehalococcoides_CBDB1 |
| Pseudoalteromonas_atlantica_T6c |
| Shewanella_W3-18-1 |
| Polynucleobacter_QLW-P1DMWA-1 |
| Anaplasma_phagocytophilum_HZ |
| Campylobacter_curvus_525_92 |
| Shewanella_denitrificans_OS217 |
| Bacillus_halodurans |
| Microcystis_aeruginosa_NIES_843 |
| Shewanella_sediminis_HAW-EB3 |
| Bacillus_amyloliquefaciens_FZB42 |
| Alteromonas_macleodii__Deep_ecotype_ |
| Vibrio_vulnificus_CMCP6 |
| Vibrio_harveyi_ATCC_BAA-1116 |
| Dehalococcoides_BAV1 |
| Coxiella_burnetii_RSA_331 |
| Shewanella_baltica_OS195 |
| Aquifex_aeolicus |
| Shewanella_baltica_OS185 |
| Syntrophomonas_wolfei_Goettingen |
| Shewanella_baltica_OS155 |
| Vibrio_vulnificus_YJ016 |
| Wolinella_succinogenes |
| Desulfitobacterium_hafniense_Y51 |
| Bacillus_clausii_KSM-K16 |
| Coxiella_burnetii |
| Yersinia_pseudotuberculosis_IP_31758 |
| Yersinia_pseudotuberculosis_PB1_ |
| Coxiella_burnetii_Dugway_7E9-12 |
| Saccharophagus_degradans_2-40 |
| Lactobacillus_brevis_ATCC_367 |
| Acaryochloris_marina_MBIC11017 |
| Yersinia_pseudotuberculosis_YPIII |
| Shewanella_oneidensis |
| Yersinia_pestis_Antiqua |
| Yersinia_pestis_Nepal516 |
| Shewanella_MR-4 |
| Shewanella_MR-7 |
| Yersinia_pseudotuberculosis_IP32953 |
| Shewanella_ANA-3 |
| Idiomarina_loihiensis_L2TR |
| Yersinia_enterocolitica_8081 |
| Synechococcus_PCC_7002 |
| Desulfotalea_psychrophila_LSv54 |
| Yersinia_pestis_Pestoides_F |
| Exiguobacterium_sibiricum_255_15 |
| Synechocystis_PCC6803 |
| Methylacidiphilum_infernorum_V4 |
| Porphyromonas_gingivalis_ATCC_33277 |
| Dehalococcoides_ethenogenes_195 |
| Thermotoga_petrophila_RKU-1 |
| Yersinia_pestis_KIM |
| Yersinia_pestis_biovar_Mediaevails |
| Thermotoga_RQ2 |
| Vibrio_cholerae_O395 |
| Yersinia_pestis_CO92 |
| Lactobacillus_casei |
| Escherichia_coli_O157H7_EDL933 |
| Thermotoga_maritima |
| Pelodictyon_phaeoclathratiforme_BU_1 |
| Escherichia_coli_536 |
| Escherichia_coli_APEC_O1 |
| Escherichia_coli_UTI89 |
| Prochlorococcus_marinus_MIT_9303 |
| Syntrophus_aciditrophicus_SB |
| Porphyromonas_gingivalis_W83 |
| Lactobacillus_casei_ATCC_334 |
| Nitrosomonas_eutropha_C71 |
| Vibrio_cholerae |
| Erwinia_carotovora_atroseptica_SCRI1043 |
| Escherichia_coli_C_ATCC_8739 |
| Escherichia_coli_W3110 |
| Escherichia_coli_SMS_3_5 |
| Yersinia_pestis_Angola |
| Lactobacillus_delbrueckii_bulgaricus_ATCC_BAA-365 |
| Escherichia_coli_O157H7 |
| Escherichia_coli_K12_substr__MG1655 |
| Escherichia_coli_CFT073 |
| Escherichia_coli_HS |
| Shewanella_loihica_PV-4 |
| Chlorobium_phaeobacteroides_DSM_266 |
| Escherichia_coli_E24377A |
| Pelotomaculum_thermopropionicum_SI |
| Geobacillus_thermodenitrificans_NG80-2 |
| Prochlorococcus_marinus_MIT9313 |
| Polynucleobacter_necessarius_STIR1 |
| Salmonella_enterica_arizonae_serovar_62_z4_z23__ |
| Enterobacter_638 |
| Lactobacillus_delbrueckii_bulgaricus |
| Chlorobium_phaeobacteroides_BS1 |
| Prosthecochloris_aestuarii_DSM_271 |
| Neisseria_gonorrhoeae_NCCP11945 |
| Salmonella_typhimurium_LT2 |
| Escherichia_coli_K_12_substr__DH10B |
| Lactobacillus_fermentum_IFO_3956 |
| Nitrosococcus_oceani_ATCC_19707 |
| Salmonella_enterica_serovar_Paratyphi_B_SPB7 |
| Citrobacter_koseri_ATCC_BAA-895 |
| Shigella_sonnei_Ss046 |
| Synechococcus_CC9311 |
| Cellvibrio_japonicus_Ueda107 |
| Neisseria_meningitidis_FAM18 |
| Bdellovibrio_bacteriovorus |
| Neisseria_meningitidis_MC58 |
| Neisseria_meningitidis_Z2491 |
| Tropheryma_whipplei_Twist |
| Nitrosomonas_europaea |
| Neisseria_meningitidis_053442 |
| Salmonella_enterica_serovar_Newport_SL254 |
| Salmonella_enterica_serovar_Heidelberg_SL476 |
| Salmonella_enterica_serovar_Schwarzengrund_CVM19633 |
| Tropheryma_whipplei_TW08_27 |
| Serratia_proteamaculans_568 |
| Xylella_fastidiosa_M23 |
| Salmonella_typhi_Ty2 |
| Salmonella_typhi |
| Prosthecochloris_vibrioformis_DSM_265 |
| Shigella_flexneri_5_8401 |
| Shigella_flexneri_2a |
| Salmonella_enterica_Paratypi_ATCC_9150 |
| Shigella_boydii_Sb227 |
| Shigella_flexneri_2a_2457T |
| Chlorobaculum_parvum_NCIB_8327 |
| Janthinobacterium_Marseille |
| Salmonella_enterica_Choleraesuis |
| Erwinia_tasmaniensis |
| Geobacter_uraniumreducens_Rf4 |
| Xylella_fastidiosa_Temecula1 |
| Xylella_fastidiosa |
| Hahella_chejuensis_KCTC_2396 |
| Shewanella_amazonensis_SB2B |
| Shigella_dysenteriae |
| Herpetosiphon_aurantiacus_ATCC_23779 |
| Herminiimonas_arsenicoxydans |
| Xylella_fastidiosa_M12 |
| Synechococcus_CC9902 |
| Corynebacterium_glutamicum_ATCC_13032_Kitasato |
| Geobacter_lovleyi_SZ |
| Shigella_boydii_CDC_3083_94 |
| Corynebacterium_glutamicum_R |
| Methylobacillus_flagellatus_KT |
| Candidatus_Desulfococcus_oleovorans_Hxd3 |
| Neisseria_gonorrhoeae_FA_1090 |
| Chlorobium_tepidum_TLS |
| Thermosynechococcus_elongatus |
| Enterobacter_sakazakii_ATCC_BAA-894 |
| Moorella_thermoacetica_ATCC_39073 |
| Nitrosospira_multiformis_ATCC_25196 |
| Pelobacter_carbinolicus |
| Akkermansia_muciniphila_ATCC_BAA_835 |
| Corynebacterium_diphtheriae |
| Klebsiella_pneumoniae_MGH_78578 |
| Anaplasma_marginale_St_Maries |
| Ochrobactrum_anthropi_ATCC_49188 |
| Magnetococcus_MC-1 |
| Heliobacterium_modesticaldum_Ice1 |
| Brucella_melitensis |
| Synechococcus_elongatus_PCC_7942 |
| Pelodictyon_luteolum_DSM_273 |
| Synechococcus_elongatus_PCC_6301 |
| Geobacter_metallireducens_GS-15 |
| Alcanivorax_borkumensis_SK2 |
| Marinobacter_aquaeolei_VT8 |
| Beijerinckia_indica_ATCC_9039 |
| Brucella_abortus_S19 |
| Synechococcus_sp_WH8102 |
| Brucella_canis_ATCC_23365 |
| Brucella_suis_1330 |
| Dechloromonas_aromatica_RCB |
| Brucella_suis_ATCC_23445 |
| Synechococcus_CC9605 |
| Aeromonas_salmonicida_A449 |
| Pelobacter_propionicus_DSM_2379 |
| Granulobacter_bethesdensis_CGDNIH1 |
| Brucella_abortus_9-941 |
| Aeromonas_hydrophila_ATCC_7966 |
| Brucella_melitensis_biovar_Abortus |
| Pseudomonas_syringae_tomato_DC3000 |
| Renibacterium_salmoninarum_ATCC_33209 |
| Sodalis_glossinidius_morsitans |
| Rhodoferax_ferrireducens_T118 |
| Agrobacterium_tumefaciens_C58_Cereon |
| Brucella_ovis |
| Polaromonas_naphthalenivorans_CJ2 |
| Cyanobacteria_bacterium_Yellowstone_B-Prime |
| Bifidobacterium_adolescentis_ATCC_15703 |
| Pseudomonas_fluorescens_PfO-1 |
| Pseudomonas_syringae_phaseolicola_1448A |
| Pseudomonas_syringae_pv_B728a |
| Candidatus_Desulforudis_audaxviator_MP104C |
| Pirellula_sp |
| Bifidobacterium_longum_DJO10A |
| Acidobacteria_bacterium_Ellin345 |
| Roseobacter_denitrificans_OCh_114 |
| Corynebacterium_jeikeium_K411 |
| Synechococcus_WH_7803 |
| Synechococcus_RCC307 |
| Treponema_pallidum |
| Treponema_pallidum_SS14 |
| Gloeobacter_violaceus |
| Geobacter_sulfurreducens |
| Chloroflexus_aurantiacus_J_10_fl |
| Gluconobacter_oxydans_621H |
| Desulfovibrio_desulfuricans_G20 |
| Cyanobacteria_bacterium_Yellowstone_A-Prime |
| Pseudomonas_putida_GB_1 |
| Pseudomonas_putida_W619 |
| Rhizobium_leguminosarum_bv_viciae_3841 |
| Mycobacterium_leprae |
| Bifidobacterium_longum |
| Pseudomonas_putida_KT2440 |
| Pseudomonas_putida_F1 |
| Rhizobium_etli_CFN_42 |
| Rhizobium_etli_CIAT_652 |
| Silicibacter_TM1040 |
| Pseudomonas_fluorescens_Pf-5 |
| Nitrobacter_winogradskyi_Nb-255 |
| Solibacter_usitatus_Ellin6076 |
| Mesorhizobium_BNC1 |
| Magnetospirillum_magneticum_AMB-1 |
| Polaromonas_JS666 |
| Corynebacterium_efficiens_YS-314 |
| Roseiflexus_RS-1 |
| Bordetella_avium_197N |
| Sinorhizobium_medicae_WSM419 |
| Propionibacterium_acnes_KPA171202 |
| Hyphomonas_neptunium_ATCC_15444 |
| Sinorhizobium_meliloti |
| Chromobacterium_violaceum |
| Pseudomonas_entomophila_L48 |
| Nitrobacter_hamburgensis_X14 |
| Mesorhizobium_loti |
| Jannaschia_CCS1 |
| Parvibaculum_lavamentivorans_DS-1 |
| Burkholderia_phymatum_STM815 |
| Corynebacterium_urealyticum_DSM_7109 |
| Ralstonia_metallidurans_CH34 |
| Chromohalobacter_salexigens_DSM_3043 |
| Erythrobacter_litoralis_HTCC2594 |
| Syntrophobacter_fumaroxidans_MPOB |
| Burkholderia_xenovorans_LB400 |
| Silicibacter_pomeroyi_DSS-3 |
| Mycobacterium_abscessus_ATCC_19977T |
| Delftia_acidovorans_SPH-1 |
| Arthrobacter_aurescens_TC1 |
| Maricaulis_maris_MCS10 |
| Pseudomonas_mendocina_ymp |
| Roseiflexus_castenholzii_DSM_13941 |
| Ralstonia_eutropha_JMP134 |
| Acidiphilium_cryptum_JF-5 |
| Pseudomonas_stutzeri_A1501 |
| Bradyrhizobium_japonicum |
| Gluconacetobacter_diazotrophicus_PAl_5 |
| Rhodospirillum_rubrum_ATCC_11170 |
| Novosphingobium_aromaticivorans_DSM_12444 |
| Methylococcus_capsulatus_Bath |
| Bordetella_petrii |
| Pseudomonas_aeruginosa_PA7 |
| Deinococcus_radiodurans |
| Pseudomonas_aeruginosa_UCBPP-PA14 |
| Desulfovibrio_vulgaris_DP4 |
| Rhodopseudomonas_palustris_BisB5 |
| Azoarcus_sp_EbN1 |
| Xanthomonas_oryzae_MAFF_311018 |
| Rhodopseudomonas_palustris_BisA53 |
| Arthrobacter_FB24 |
| Bradyrhizobium_BTAi1 |
| Rhodopseudomonas_palustris_TIE_1 |
| Pseudomonas_aeruginosa |
| Acidovorax_JS42 |
| Thiobacillus_denitrificans_ATCC_25259 |
| Ralstonia_eutropha_H16 |
| Rhodopseudomonas_palustris_CGA009 |
| Xanthomonas_oryzae_KACC10331 |
| Sphingomonas_wittichii_RW1 |
| Desulfovibrio_vulgaris_Hildenborough |
| Sphingopyxis_alaskensis_RB2256 |
| Dinoroseobacter_shibae_DFL_12 |
| Bradyrhizobium_ORS278 |
| Burkholderia_383 |
| Salinibacter_ruber_DSM_13855 |
| Burkholderia_cenocepacia_MC0_3 |
| Rhodopseudomonas_palustris_BisB18 |
| Xanthomonas_campestris_8004 |
| Xanthomonas_campestris_vesicatoria_85-10 |
| Burkholderia_ambifaria_MC40_6 |
| Xanthomonas_campestris |
| Burkholderia_cenocepacia_HI2424 |
| Mycobacterium_marinum_M |
| Burkholderia_cepacia_AMMD |
| Caulobacter_K31 |
| Burkholderia_vietnamiensis_G4 |
| Xanthomonas_citri |
| Verminephrobacter_eiseniae_EF01-2 |
| Mycobacterium_tuberculosis_F11 |
| Cupriavidus_taiwanensis |
| Mycobacterium_tuberculosis_H37Rv |
| Caulobacter_crescentus |
| Ralstonia_solanacearum |
| Deinococcus_geothermalis_DSM_11300 |
| Bordetella_bronchiseptica |
| Mycobacterium_bovis_BCG_Pasteur_1173P2 |
| Paracoccus_denitrificans_PD1222 |
| Burkholderia_cenocepacia_AU_1054 |
| Mycobacterium_bovis |
| Mycobacterium_tuberculosis_H37Ra |
| Rhodopseudomonas_palustris_HaA2 |
| Mycobacterium_smegmatis_MC2_155 |
| Stenotrophomonas_maltophilia_K279a |
| Rhodococcus_RHA1 |
| Mycobacterium_gilvum_PYR-GCK |
| Burkholderia_multivorans_ATCC_17616_Tohoku |
| Stenotrophomonas_maltophilia_R551_3 |
| Mycobacterium_JLS |
| Mycobacterium_vanbaalenii_PYR-1 |
| Mycobacterium_KMS |
| Mycobacterium_MCS |
| Leptothrix_cholodnii_SP_6 |
| Methylobacterium_extorquens_PA1 |
| Thermobifida_fusca_YX |
| Azorhizobium_caulinodans_ORS_571 |
| Mycobacterium_tuberculosis_CDC1551 |
| Xanthobacter_autotrophicus_Py2 |
| Acidovorax_avenae_citrulli_AAC00-1 |
| Azoarcus_BH72 |
| Halorhodospira_halophila_SL1 |
| Alkalilimnicola_ehrlichei_MLHE-1 |
| Opitutus_terrae_PB90_1 |
| Symbiobacterium_thermophilum_IAM14863 |
| Burkholderia_thailandensis_E264 |
| Rubrobacter_xylanophilus_DSM_9941 |
| Thermus_thermophilus_HB27 |
| Mycobacterium_ulcerans_Agy99 |
| Burkholderia_pseudomallei_1710b |
| Mycobacterium_avium_paratuberculosis |
| Burkholderia_pseudomallei_1106a |
| Thermus_thermophilus_HB8 |
| Rhodobacter_sphaeroides_ATCC_17025 |
| Burkholderia_pseudomallei_668 |
| Bordetella_pertussis |
| Bordetella_parapertussis |
| Mycobacterium_avium_104 |
| Leifsonia_xyli_xyli_CTCB0 |
| Methylibium_petroleiphilum_PM1 |
| Burkholderia_mallei_NCTC_10247 |
| Myxococcus_xanthus_DK_1622 |
| Burkholderia_mallei_NCTC_10229 |
| Burkholderia_mallei_SAVP1 |
| Burkholderia_pseudomallei_K96243 |
| Salinispora_tropica_CNB-440 |
| Nocardia_farcinica_IFM10152 |
| Salinispora_arenicola_CNS-205 |
| Rhodobacter_sphaeroides_ATCC_17029 |
| Rhodobacter_sphaeroides_2_4_1 |
| Frankia_CcI3 |
| Burkholderia_mallei_ATCC_23344 |
| Streptomyces_avermitilis |
| Acidothermus_cellulolyticus_11B |
| Methylobacterium_radiotolerans_JCM_2831 |
| Saccharopolyspora_erythraea_NRRL_2338 |
| Nocardioides_JS614 |
| Frankia_EAN1pec |
| Methylobacterium_4_46 |
| Streptomyces_coelicolor |
| Sorangium_cellulosum__So_ce_56_ |
| Clavibacter_michiganensis_NCPPB_382 |
| Streptomyces_griseus_NBRC_13350 |
| Kocuria_rhizophila_DC2201 |
| Frankia_alni_ACN14a |
| Clavibacter_michiganensis_sepedonicus |
| Kineococcus_radiotolerans_SRS30216 |
| Anaeromyxobacter_Fw109-5 |
| Anaeromyxobacter_dehalogenans_2CP-C |
| Lactobacillus_reuteri_F275_JGI |
| Bacillus_licheniformis_ATCC_14580 |
| Escherichia_coli_SECEC_SMS_3_5 |
| Corynebacterium_glutamicum_ATCC_13032_Bielefeld |
| Burkholderia_multivorans_ATCC_17616_JGI |
