# Supplementary material for: General Rules for Optimal Codon Choice
Source: PLoS Genet. 2009 Jul 10;5(7):e1000556. doi: 10.1371/journal.pgen.1000556 (PMC2700274; doi:10.1371/journal.pgen.1000556)
Supplement: Text S2 — Fungi used in this study. (0.04 MB DOC) [file pgen.1000556.s008.doc]

**Text S2.** Fungi used in this study

| Candida_glabrata_CBS138 |
| --- |
| Cryptococcus_neoformans_var_JEC21 |
| Debarymoymyces_hansenii_CBS767 |
| Encephalitozoon_cuniculi |
| Eremothecium_gossypii |
| Kluyveromyces_lactis_NRPL_Y-1140 |
| Pichia_stipitis |
| Saccharomyces_cerevisiae |
| Schizosaccharomyces_pombe |
| Yarrowia_lipolytica_CLIB122 |
