# Supplementary material for: General Rules for Optimal Codon Choice
Source: PLoS Genet. 2009 Jul 10;5(7):e1000556. doi: 10.1371/journal.pgen.1000556 (PMC2700274; doi:10.1371/journal.pgen.1000556)
Supplement: Text S3 — Archea used in this study. (0.08 MB DOC) [file pgen.1000556.s009.doc]

**Text S3.** Archea used in this study

| Methanosphaera_stadtmanae |
| --- |
| Methanobrevibacter_smithii_ATCC_35061 |
| Methanococcus_aeolicus_Nankai-3 |
| Picrophilus_torridus_DSM_9790 |
| Methanococcus_maripaludis_S2 |
| Methanococcus_vannielii_SB |
| Methanococcus_maripaludis_C6 |
| Methanococcus_maripaludis_C5 |
| Methanococcus_maripaludis_C7 |
| Methanococcus_jannaschii |
| Sulfolobus_tokodaii |
| Nitrosopumilus_maritimus_SCM1 |
| Methanosarcina_barkeri_fusaro |
| Sulfolobus_solfataricus |
| Sulfolobus_acidocaldarius_DSM_639 |
| Thermoplasma_volcanium |
| Methanosarcina_mazei |
| Staphylothermus_marinus_F1 |
| Methanococcoides_burtonii_DSM_6242 |
| Methanosarcina_acetivorans |
| Pyrococcus_furiosus |
| Thermoplasma_acidophilum |
| Caldivirga_maquilingensis_IC-167 |
| Pyrococcus_horikoshii |
| Nanoarchaeum_equitans |
| Methanospirillum_hungatei_JF-1 |
| Metallosphaera_sedula_DSM_5348 |
| Pyrococcus_abyssi |
| Methanobacterium_thermoautotrophicum |
| Archaeoglobus_fulgidus |
| Methanocorpusculum_labreanum_Z |
| Thermococcus_kodakaraensis_KOD1 |
| Candidatus_Korarchaeum_cryptofilum_OPF8 |
| Haloquadratum_walsbyi |
| uncultured_methanogenic_archaeon_RC-I |
| Pyrobaculum_aerophilum |
| Candidatus_Methanoregula_boonei_6A8 |
| Methanosaeta_thermophila_PT |
| Pyrobaculum_arsenaticum_DSM_13514 |
| Pyrobaculum_islandicum_DSM_4184 |
| Aeropyrum_pernix |
| Hyperthermus_butylicus |
| Pyrobaculum_calidifontis_JCM_11548 |
| Thermofilum_pendens_Hrk_5 |
| Haloarcula_marismortui_ATCC_43049 |
| Ignicoccus_hospitalis_KIN4_I |
| Methanoculleus_marisnigri_JR1 |
| Thermoproteus_neutrophilus_V24Sta |
| Natronomonas_pharaonis |
| Halobacterium_salinarum_R1 |
| Halobacterium_sp |
| Methanopyrus_kandleri |
